# Evaluating the integration of eye-tracking and motion capture technologies: Quantifying the accuracy and precision of gaze measures

**DOI:** 10.1177/20416695221116652

**Published:** 2022-09-26

**Authors:** Rhys Hunt, Tim Blackmore, Chris Mills, Matt Dicks

**Affiliations:** School of Sport, Health and Exercise Science, 6697 University of Portsmouth, Portsmouth, UK

**Keywords:** gaze behavior, eye tracking, motion capture

## Abstract

Integrating mobile eye tracking and optoelectronic motion capture enables point of gaze to be expressed within the laboratory co-ordinate system and presents a method not commonly applied during research examining dynamic behaviors, such as locomotion. This paper examines the quality of gaze data collected through the integration. Based on research suggesting increased viewing distances are associated with reduced data quality; the accuracy and precision of gaze data as participants (*N* = 11) viewed floor-based targets at distances of 1–6 m was investigated. A mean accuracy of 2.55 ± 1.12° was identified, however, accuracy and precision measures (relative to targets) were significantly (*p* < .05) reduced at greater viewing distances. We then consider if signal processing techniques may improve accuracy and precision, and overcome issues associated with missing data. A 4th-order Butterworth lowpass filter with cut-off frequencies determined via autocorrelation did not significantly improve data quality, however, interpolation via Quintic spline was sufficient to overcome gaps of up to 0.1 s. We conclude the integration of gaze and motion capture presents a viable methodology in the study of human behavior and presents advantages for data collection, treatment, and analysis. We provide considerations for the collection, analysis, and treatment of gaze data that may help inform future methodological decisions.

## Introduction

The past two decades have seen a notable increase in the development of research methods incorporating mobile eye tracking technologies to examine the visual control of behavior across a variety of scientific domains (Tatler et al., 2011). By way of example, methods used in the study of social attention (Laidlaw et al., 2011) and expertise in sport (Vickers, 1996) have been enriched, leading to new proposals of dynamic visual control, which consider the function of eye movements relative to the coordination of the head and body (van der Kamp & Dicks, 2017). Within the field of perceptual-motor control, the accurate study of coordination between the eyes, head, and body has arguably been limited by the lack of suitable technology (Kothari et al., 2020). In such fields, researchers using head-worn eye trackers are predominantly interested in questions such as which objects in the world a person fixated, in what order, and for how long (Ellmers et al., 2020; Matthis et al., 2018; Niehorster, Hessels, et al., 2020). As such, eye movement data collected via head mounted eye tracking technology requires mapping onto locations in world space (Hessels et al., 2018). While mobile eye trackers deliver gaze data in a head-centered reference frame, simultaneous motion capture provides the means to express gaze data in a world-centered reference frame. Specifically, by combining eye position, derived from retroreflective markers attached to the mobile eye tracker, and eye orientation data, point of gaze can be expressed relative to the world-space coordinate system.^1^

Although the integration of mobile eye tracking and motion capture systems to express point of gaze with a world-centered frame of reference is uncommon (however, see: Burger et al., 2018; Essig et al., 2012; Matthis et al., 2018), research capturing kinematic and gaze data has begun to progress our understanding of perceptual-motor behaviors (Domínguez-Zamora et al., 2018; Domínguez-Zamora & Marigold, 2021). In particular, Matthis et al. (2018) recently developed a method of integrating gaze and kinematic measures, expressing point of gaze within world-space whilst participants walked over terrains of differing complexity. Deriving eye and horizontal floor plane position from inertial measurement unit (IMU) sensors located on each participant's head and feet, and gaze orientation data from a mobile eye tracker, point of gaze was computed as the location where the gaze vector intercepted the floor plane^2^. Matthis and colleagues’ method developed understanding of how people successfully overcome challenging environments, revealing that gaze and kinematic behaviors are adapted relative to the environment's specific demands. As such, their method signposted the potential benefits of integrating these distinct measurement systems in perceptual-motor control research domains requiring skillful coordination, such as locomotion (Higuchi, 2013; Matthis et al., 2018) and sport performance (Dicks et al., 2010), as well as domains such as environmental representation (Tatler & Tatler, 2013) and object manipulation (Draschkow & Võ, 2016; van Dijk & Bongers, 2014).

Although data collected using motion capture and eye tracking systems have been integrated previously (Burger et al., 2018; Jantunen et al., 2020; Matthis et al., 2018) the quality of data, such as point of gaze accuracy and precision, in terms of world-space co-ordinates has yet to be evaluated. As such, this paper centralizes around accuracy, defined as the difference between the recorded gaze position and the actual gaze position (Wang et al., 2017), and precision, defined as the ability to reliably reproduce a measurement given a fixating eye (Pastel et al., 2020) of data collected through the integration of mobile eye tracking and optoelectronic motion capture and then expressed in world-space co-ordinates.

### Evaluating Optoelectronic Systems for Gaze Data Collection

When precise position or orientation-related analyses of human movements are required, optoelectronic systems represent the gold-standard in terms of accurately tracking (<1 mm) anatomical positions (Spörri et al., 2016; van der Kruk & Reijne, 2018). Determining the position of the eye is essential to accurately expressing point of gaze in relation to the world co-ordinate system, therefore, utilizing optoelectronic motion capture may enhance gaze data quality compared to alterative motion capture systems. Moreover, unlike IMU, optoelectronic systems allow features such as objects, obstacles, or foot targets to be digitized, and expressed within the world-space coordinate system (van der Kruk & Reijne, 2018; Matthis et al., 2018).

The ability to accurately translate point of gaze onto the environment may play a pivotal role in analyzing gaze data. Traditionally, gaze data analysis has been undertaken by frame-by-frame analysis where researchers manually classify fixations or saccades and then identify the locations participants fixate (Vansteenkiste et al., 2014). More recently, event-based analysis has streamlined this analysis process by using algorithms to classify gaze events based on eye orientation data (Benjamins et al., 2018; Hessels et al., 2017; Niehorster, Hessels, et al., 2020). However, these algorithm based approaches are limited when evaluating gaze behaviors in locomotor settings as classifying gaze behaviors based exclusively on eye orientation may not account for disparity between the participants eye and bodily motion (Hessels et al., 2018; Lappi, 2015). As such, as a person moves through the environment, determining point of gaze relative to environmental features has predominantly been achieved by frame-by-frame analysis of scene camera footage (Hessels, Benjamins, et al., 2020; Jongerius et al., 2021; Kothari et al., 2020). In particular, a frame-by-frame approach has been commonly applied to consider the amount of time a person's point of gaze is located on designated environmental locations (Ellmers et al., 2016, 2020; Mele & Federici, 2012; Parr et al., 2020). However, it has been suggested that frame-by-frame analysis methods are prone to inter-observer error and have been considered time consuming (Duchowski, 2007; Hessels, Niehorster, et al., 2020; Kiefer et al., 2017). Further, researchers evaluating eye-tracking data analysis have recently noted that eye-tracking research would benefit from more standardized analysis procedures to enhance comparison between studies and assessment of study quality (Jongerius et al., 2021). The capacity of optoelectronic data collection to express both point of gaze and the location of environmental features within a world-centered reference frame addresses these points by facilitating greater automation of the data analysis process, which promotes time efficiency, reduces inter-observer error, and greatly increases the number of trials that can be analyzed. In turn, these attributes increase statistical power, which due to the time needed to manually code gaze data has been frequently recognized as low (Jongerius et al., 2021; Knudson, 2017). In addition to the advantages associated with automated data analysis, the ability to express both point of gaze and environmental features within the laboratory coordinate system allows researchers to evaluate the accuracy and precision of gaze data in terms of world-space and contributes to effective experimental design.

### Gaze Data Accuracy and Precision

**Figure 1.**
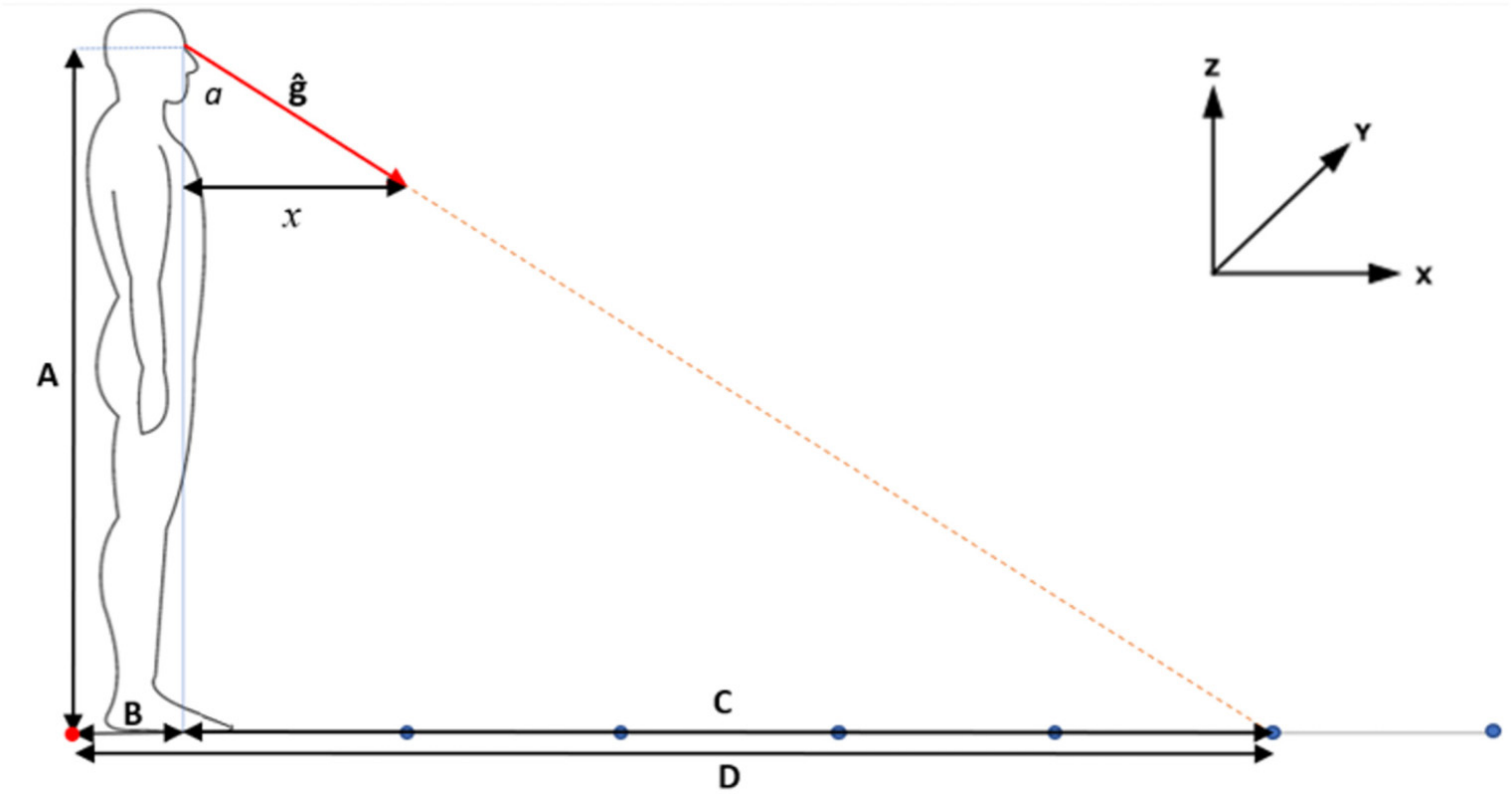
Computation of *x* axis floor intercept location. Red dot represents lab origin; blue dots represent floor markers positioned along the ground plane (*z* = 0); red arrow represents gaze unit vector *ĝ*; orange dotted line extrapolated gaze vector included to clarify intercept location; *x* represents the gaze vectors *x* axis magnitude; Angle *a* represents the viewing angle from vertical plane; length A represents eye *z* axis position; length B represents eye *z* axis position; length C represents look-ahead distance; length D represents *x* axis intercept distance.

Aside from establishing the origin of the gaze vector (e.g., eye position), expressing point of gaze with a world-based frame of reference can be affected by the accuracy and precision of the eye tracking system (Ooms, 2015). Multiple factors influence gaze calibration and data quality, including task factors such as viewing distance defined as the distance between a participant and the location being fixated (such as Figure 1 ‘C’) and viewing angle defined as the angle between gaze origin and the location being fixated (such as Figure 1 ‘*a*’) (Blignaut & Wium, 2014; Thibeault et al., 2019). Parallax error represents a common limitation associated with viewing distance and head-mounted eye-trackers, which occurs when the distance between the point of regard and the user (viewing distance) is different to when the system was calibrated. Because the scene camera and the eye are not co-axial, viewing distance and angle affect the mapping between point of gaze and scene-camera footage (Mardanbegi & Hansen, 2012). However, by establishing the position of the eye via optoelectronic motion capture (e.g., mapping the eye position onto the world-space coordinate system rather than in relation to head-based camera footage), the spatial offset between eye and scene-camera should potentially alleviate parallax error. That said, although using motion capture to translate gaze data from head to world may lessen parallax error, issues may remain in data quality as a function of task factors, such as high-speed movement though world space, or marker occlusion (Spörri et al., 2016).

MacInnes et al. (2018) considered the influence of viewing distance and angle by evaluating the quality of gaze data at distances of 1, 2, and 3 m and lateral angles of −10°, 0°, and 10°, reporting the mean accuracy of the Tobii Pro Glasses 2 system across the aforementioned conditions as 1.42° of visual angle. Although accuracy may be expressed in terms of visual angle (e.g., 1.42°), it is important to consider the effects the relationship between visual angle and viewing distance may have on accuracy when considering a world-based reference frame. For example, adaptive locomotor research indicates that visual information is exploited from distances within approximately 6 m preceding a foot target or obstacle (Cornus et al., 2009; Lee et al., 1982; Montagne et al., 2000; van Andel et al., 2018). Assuming a constant accuracy value of 1.42°, viewing an eye-height target located on a vertical plane (such as a wall) from a distance of 1 m translates to a world-space accuracy of 2.5 cm, whereas a viewing distance of 6 m translates to an accuracy of 14.9 cm. As such, an increase in viewing distance would lead to reduced world-space accuracy due to constant angles covering larger areas at larger distances.

During everyday locomotion people predominantly attend to task relevant ground-based locations in the lower visual field (Matthis et al., 2018; Marigold & Patla, 2008). When considering the world-space accuracy of ground-based fixations, the angle of incidence must be considered alongside viewing distance. Assuming a constant eye height and constant accuracy (in visual angle), the distance along the horizontal plane increases non-linearly with viewing distance. For example, a fixed eye height of 1.57 m (Adler, 1999) and an accuracy of 1.42° is associated with a distance along the horizontal plane of approximately 5.5 cm for a person fixating a floor based target, 1 m ahead of their location. However, accuracy would decrease to 60.8 cm when fixating a floor-based target that is located at a distance 6 m away.

Although the combined influence of viewing distance and visual angle outlined above are simple to translate into world-space values using trigonometry, the accuracy of eye-tracking systems (in terms of visual angle) has also been shown to be influenced by a range of participant factors such as eye color, contact lens use, and eye lash length (e.g., Blignaut & Wium, 2014; Thibeault, et al., 2019). Furthermore, MacInnes et al. (2018) also reported that accuracy decreased as viewing distance increased. Specifically, visual angles of 0.8, 1.6, and 1.8° were reported for distances of 1, 2, and 3 m, respectively. The fact that viewing distance and participant factors impacted eye tracking accuracy (MacInnes et al., 2018; Pastel et al., 2020) raises important considerations when measuring gaze behaviors throughout situations such as adaptive locomotion, where attending to distal information has been noted as vital (Higuchi, 2013). Additionally, MacInnes and colleagues reported reduced precision when viewing angle increased, a result possibly associated with the location of the pupils relative to the eye-tracking system's cameras (Hornof & Halverson, 2002; Pastel et al., 2020). This is again an important consideration as a participant fixating a proximal ground-based location would exhibit greater viewing angles compared to when fixating a target positioned at eye height, possibly reducing gaze data quality (MacInnes et al., 2018). Such findings indicate that world-space accuracy may not be wholly defined geometrically and establishes a need to consider the influence of viewing distances and angles on gaze data quality in terms of world-space co-ordinates when viewing floor-based locations.

### Processing Gaze Data

Noise has commonly been acknowledged as a limitation with kinematic data (Camargo et al., 2020; Winter, 2009) and is particularly prevalent with optoelectronic data collection methods. As optoelectronic motion capture provides the information required to translate point of gaze into the world-centered reference frame, data processing methods commonly employed to overcome noise may improve gaze data quality and accuracy. To reduce the influence of noise, and improve data quality, researchers have commonly applied a low-pass Butterworth filter to data collected via optoelectronic motion capture (Parr et al., 2020; Spörri et al., 2016; van der Kruk & Reijne, 2018). Accordingly, filtering eye position data may improve data quality and accuracy when translating gaze position to a world-centered reference frame.

In eye-tracking data, noise has also been identified as a property inherent to the measurement device (Niehorster, Zemblys, et al., 2020). Similar to treating kinematic data, previous researchers have utilized a low-pass Butterworth filter to treat gaze data during locomotor pointing tasks (Domínguez-Zamora et al., 2018), or to smooth gaze data prior to event classification (Houpt et al., 2018). However, these approaches commonly apply standardized one-size fits all filter parameters to gaze datasets. Such an approach may not reflect the variable nature of gaze behaviors that are common both between and within participants (Dicks et al., 2017). In recognition of the individual nature of gaze data, an auto-correlation approach to determining the optimum low-pass filter parameters (Challis, 1999) may be particularly beneficial and allow filter parameters to be objectively determined on a trial-by-trial basis (Davis & Challis, 2020).

Missing data has been acknowledged as a limitation of data collected through both optoelectronic motion capture (e.g., marker occlusion) and eye tracking (e.g., pupil identification loss; Blignaut & Wium, 2014; Duchowski, 2007; Hessels et al., 2015; Spörri et al., 2016). Across a range of disciplines, including eye tracking research, the use of splines has been commonly advocated as a simple yet robust method to interpolate missing data (Frank et al., 2009; Hessels et al., 2017; Howarth & Callaghan, 2010; Kamali et al., 2020). In particular, quintic, compared to cubic, splines have been recognized as capable of interpolating complex or variable movements (Grimshaw et al., 2019; Winter, 2009), qualities often noted within gaze data sets (Dicks et al., 2017). However, diminished accuracy has been identified as a limitation of treating larger gaps using splines (Howarth & Callaghan, 2010). To mediate this limitation, an upper gap size threshold of 100 ms has been adopted when applying splines to interpolate gaze data (Frank et al., 2009; Hessels et al., 2017). However, as considered, a standardized one-size fits all gap size threshold parameter may not be best suited to treating variable gaze behaviors (Dicks et al., 2017). As such, the use of splines as a method of filling different sized gaps in gaze data, relative to world-space accuracy merits exploration.

While mobile eye trackers deliver gaze data in a head-centered reference frame for many research topics, such as adaptive locomotion, gaze data in a world-centered reference frame is considered beneficial (Matthis et al., 2018). One way to achieve this translation is to perform simultaneous motion capture of the participant's eye position, which provides the information required to perform the head to world transformation. Findings concerning eye-tracking data have alluded to task and participant factors that inhibit gaze data quality (Blignaut & Wium, 2014). Such findings establish a need to consider the influence of greater viewing distances and viewing angles on gaze data quality in terms of world space co-ordinates. As such, this study primarily aimed to assess the accuracy and precision of eye tracking data collected using the integrated Tobii Pro Glasses 2 and Qualisys motion capture systems. Based on the importance of ground-based fixations throughout many activities of daily life, including locomotion, gaze data accuracy and precision shall be considered as participants attend to floor-based targets at distances between 1 and 6 m. Building on previous work highlighting reductions in gaze data accuracy at greater distances and viewing angles (MacInnes et al., 2018), we hypothesize a reduction in accuracy when participants attended to the most proximal and distal floor targets (1 and 6 m, respectively). Issues such as noise and missing data have also been identified in both optoelectronic and eye tracking data sets (Camargo et al., 2020; Hessels et al., 2017). Subsequently, our second aim was to explore if signal processing techniques, such as filtering and interpolation, may help overcome such limitations and improve data quality when considering gaze data in terms of world space co-ordinates.

## Methods

### Participants

G*Power (Faul et al., 2007) was used to perform an *a priori* power analysis for a repeated-measures within factors ANOVA selected to detect differences in accuracy and precision measurements as a single group of participants attended to six ground-based targets. The sample size required was calculated based on Cohen's guidelines (Cohen, 1988). The power analysis suggested that a sample size of 11 was required to detect a difference between the six ground-based target conditions with 80% probability. Following institutional ethical approval, 11 participants (*M* age = 29.36 ± 4.51; Male = 9) provided ethical consent and completed a single data collection. Consistent with previous research, a Snellen eye test established that no participant had deficits in visual acuity, with each participant scoring 20/40 vision or better (Young et al., 2012).

### Hardware

A Tobii Pro Glasses 2 mobile eye tracker (Tobii, 2018) was used to measure gaze behavior. The eye tracker consists of a lightweight (45 g) fixed geometry frame containing four cameras, and 12 illuminators, which project near infrared light to create a pattern on the cornea of both eyes. The system allows the position of each eye to be recorded at 50 Hz as per recommendations for research concerning larger viewing angles, such as the current study (Tobii, 2018). The glasses were connected to a portable recording unit which used image-processing algorithms and a physiological 3D model of the eye to estimate the position of the pupil relative to the glasses^3^. The recording unit allowed gaze data to be streamed real-time via Wi-Fi and interfaced with the Qualisys motion capture interface (QTM version, 2018.1).

**Figure 2.**
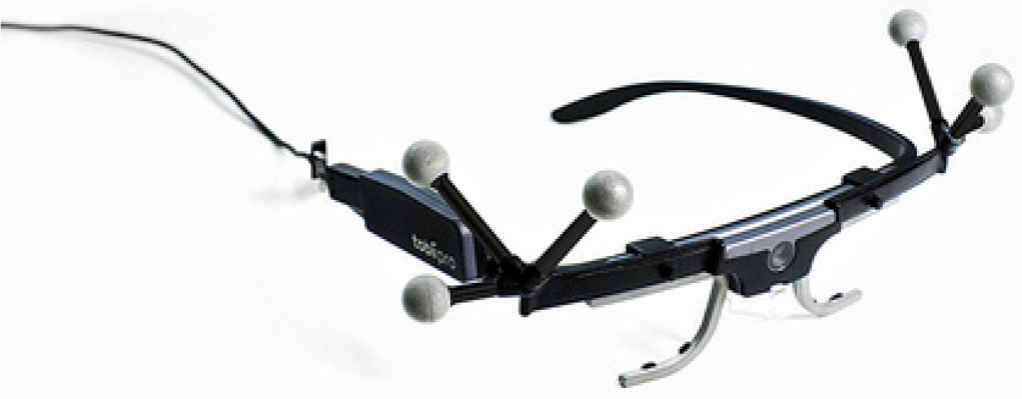
Tobii Pro glasses 2 with motion capture markers attached (Tobii, 2018).

To track the origin of each eye's gaze vector in the lab, a cluster marker (provided by Tobii) set comprised of six retroreflective markers was attached to the fixed geometry body of the eye tacking glasses (Figure 2). Within Qualisys track manager (QTM) software, a 6 degrees of freedom model was created which defined the position of each eye relative to the marker set, in turn allowing the location of each eye to be expressed relative to the lab coordinate system (QTM, version 2018.1).

### Laboratory Environment

A 14-camera system (Qualisys Oqus 300 + ) set to capture at 100 Hz was used to record the position of the eye tracker cluster marker set. In order to account for variations in participant stature, cameras were typically positioned further back from the calibrated area and focused on the upper body. An area (approximately 6 × 1 × 2 m, *x* (anterior–posterior), *y* (medial–-lateral), *z* (vertical) dimensions, respectively) was calibrated with a mean marker deviation upper limit of 1.48 mm being adopted based on average errors reported in multicamera photogrammetry literature (Summan et al., 2015).

### Procedures and Data Collection

**Figure 3.**
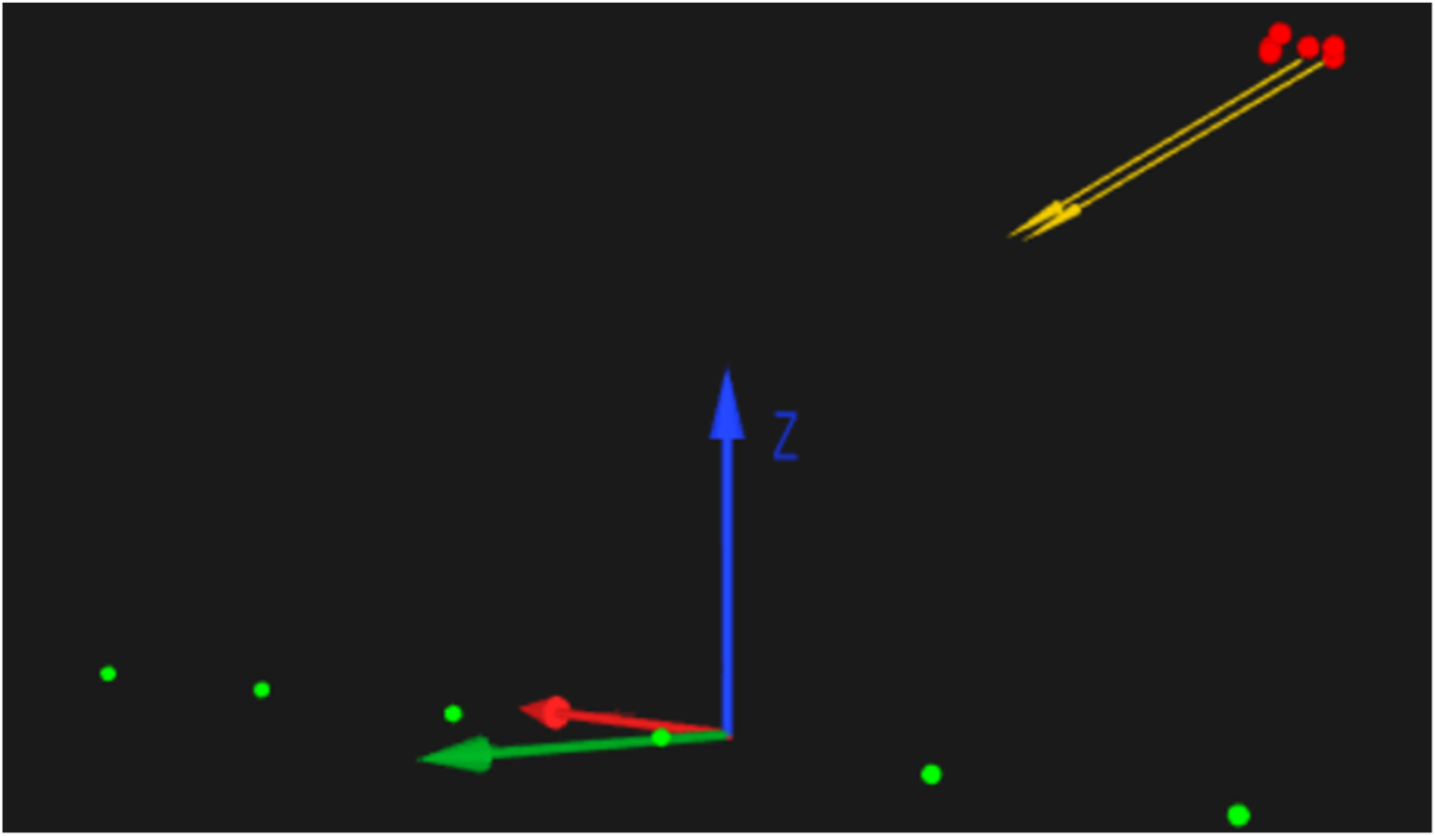
Gaze vector (yellow), retroreflective markers (red), and axis orientation visible in the QTM environment (*x*, *y*, *z* axis denoted by red, green, and blue arrows, respectively). Green dots show the floor-based target positioned at 1 m intervals along the *x* axis between 1 m (floor target 1, extreme right) and 6 m (floor target 6, extreme left) from the participant's position.

Participants were asked to tie back long hair and remove eye make-up before the eye tracker was fitted and secured in place using a head strap to reduce movement and ensure a fixed relationship between the marker set and eye position in lab space. The eye tracker was calibrated following Tobii's one-point calibration method. Following initial calibration, data were collected directly though the QTM interface with the gaze vector position visible in real time (Figure 3). Following the steps outlined by MacInnes et al. (2018), viewing the real-time gaze vector position allowed researchers to judge calibration accuracy by asking participants to fixate on prominent eye-height features, such as the motion capture cameras. Large discrepancies in vector position, such as divergent or erratic gaze vectors, resulted in the glasses being refitted and the calibration process repeated until accuracy was considered acceptable.

Following calibration, participants completed a single 30 s (1500 sample) data collection participants were instructed to remain standing in a designated location whilst fixating on six ground-based retroreflective markers (Target 1–6) positioned at 1 m intervals along the *x* axis from the participant's standing position (Figure 3). Participants were instructed to fixate each target for approximately 2 s, moving from target to target sequentially for the whole 30 s (1500 sample) period. During the data collection participants head and body movement was not constrained.

Data from each eye was collected separately, and exported to MATLAB (MathWorks Inc, 2019) for processing, which allowed gaze accuracy and precision measures to be evaluated across 22 data sets (i.e., one data set per eye). The export consisted of a matrix of six rows and a column per sample of data collection. The first three rows represented the origin position within the lab coordinate system and the second three rows represented gaze orientation as the dimensions of a unit vector.

### Calculating Gaze Location

Three distinct elements are required to be accurately measured to compute the gaze vector's intercept with the laboratory environment. These are the origin of the gaze vector, comprising of the coordinates of the gaze vector origin (e.g., eye position), gaze direction, and information about world layout, such as areas of interest, specified within the lab coordinate system. This section details the process for generating coordinates for the intercept of the gaze vectors with the floor, as specified as having a *Z* axis value (vertical height) of zero (Figure 3). Throughout this study the intercept along the *x* (anterior–posterior) axis was evaluated. This was due to the linear nature of locomotor pointing tasks, frequently examined throughout perceptual-motor control research (Ellmers et al., 2020; Hildebrandt & Cañal-Bruland, 2020; van Andel et al., 2018) and the current study's aim to evaluate data quality relative to viewing distance.^4^

First, the angle of the gaze vector relative to the vertical plane was calculated (Figure 1, *a*). Because the gaze orientation was expressed in the dimensions of a unit vector (Figure 1, ĝ) knowledge of both the distance in the *x* direction (Figure 1, *x*) and the vector magnitude allowed the viewing angle (Figure 1, *a*) to be computed using trigonometry. Next, the look-ahead distance (Figure 1, C) was calculated using the vertical eye position (Figure 1, A) and the viewing angle. Finally, the floor intercept relative to the lab coordinate system (Figure 1, D) was calculated through summing the eye's position in the *x* axis (Figure 1, B) and the look-ahead distance (Figure 1, C). This procedure was repeated for each sample of capture using a custom MATLAB script.

### Measures and Analyses

**Figure 4.**
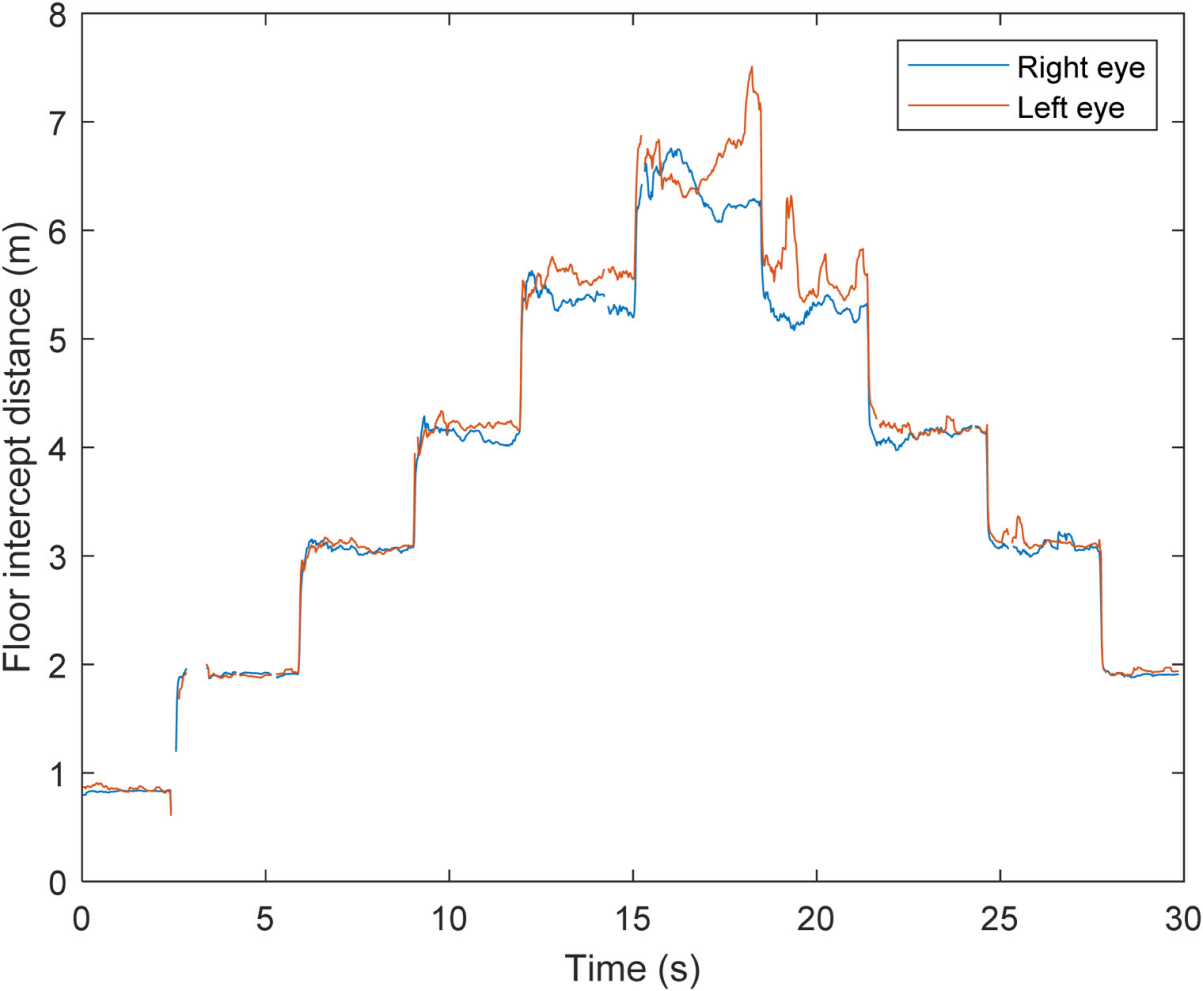
Untreated gaze data for the left and right eye (blue and red lines, respectively) from one participant when looking at the six different floor targets.

Using a custom MATLAB script which identified peaks in point of gaze velocity, the gaze data that corresponded with the start and end of each floor target viewing period was identified. These viewing periods were then manually confirmed though comparison to a plot of each participant's point of gaze, prior to data treatment (e.g., Figure 4). This approach allowed for the gaze transfer between targets (vertical lines, Figure 4) to be omitted from subsequent analysis. The identified samples (that correspond to each floor target viewing period) were then used to allow the gaze floor intercept location to be analyzed relative to the target location.

To screen gaze data for inaccuracies and consistent with approaches applied to event classification filters (Duchowski et al., 2002), the viewing angle of each eye, in relation to the *z* axis (Figure 1, *a*), was calculated in degrees from the imported unit vectors. Using MATLAB (MathWorks Inc, 2019), the angular velocity of each eye when attending to floor targets, was assessed against an angular velocity upper limit. This threshold was extrapolated from research establishing the velocity of vertical saccades is relational to angular magnitude (Collewijn et al., 1988). Specifically, based on the A-P displacement between floor targets at 1 and 2 (as associated with peak change in viewing angle) and participant eye height, a maximum amplitude between floor targets of 19.4° was identified. Collewijn and colleagues suggested that the maximum speed of upwards and downward saccades (which is comparable to looking from floor targets 1 to 2, and back from targets 2 to 1) was consistent for amplitudes of up to 30° and presented peak velocities of approximately 350°/s for saccades of 20°. Data from one participant's left eye only exceeded this value (peak 373°/s) and thus was excluded from subsequent analysis.^5^

#### Accuracy

The mean absolute error (MAE) was computed for each eye on a trial-by-trial basis. The distance between point of gaze and the floor target being fixated was computed for each sample, summed, then divided by the number of samples spent viewing the respective target (see Dietzsch et al., 2017). MAE was computed for each eye and then averaged and used for statistical analysis (Chai & Draxler, 2014; Willmott & Matsuura, 2005). As accuracy is commonly expressed in eye tracking literature as the visual angle (in degrees) between the point of gaze and the target location (Holmqvist et al., 2010; MacInnes et al., 2018), the visual angle associated with each floor-based target's MAE was computed as follows:

$$Visual\;angle=\tan^{-1}\;\left(\frac{Look\;ahead\;distance\;+\left(\frac{MAE}{2}\;\right)}{Origin\;eye\;height}\right)-\tan^{-1}\;\left(\frac{Look\;ahead\;distance-\left(\frac{MAE}{2}\;\right)}{Origin\;eye\;height}\right)$$

The mean visual angle was computed from the data of both eyes to give a measure of accuracy associated with fixations on the floor-based targets and used to compare between distances.

#### Precision

In line with extant literature (Niehorster, Zemblys, et al., 2020), precision of gaze position data was measured using the root mean square of the displacement between successive gaze position samples (RMS-S2S) and the standard deviation of the gaze position samples (STD). As such, both RMS-S2S and STD were calculated to measure precision for each of the identified fixation targets following the methods outlined in previous research (Niehorster, Zemblys et al., 2020). These values were calculated for each eye on a trial-by-trial basis with a world-based frame of reference.^6^

### Signal Processing

**Figure 5.**
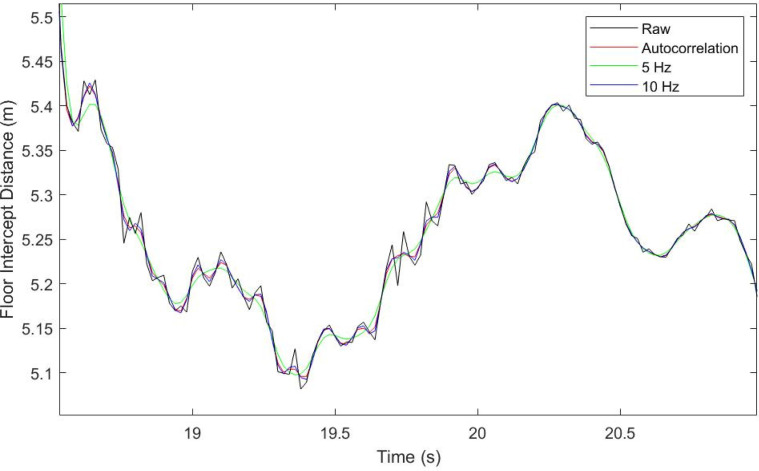
Visual representation of different filter parameters treating the same data as presented in Figure 5. Black line shows raw, untreated, data; red line shows data filtered using the autocorrelation procedure; green and blue show data treated with filter parameters of 5 and 10 Hz, respectively.

To consider the effect of signal processing on the resultant point of gaze, gaze origin and viewing angle data were filtered separately using a lowpass 4th order Butterworth filter, at the cut-off frequency determined using autocorrelation (Challis, 1999). The autocorrelation function identified a range of frequencies between 5 and 10 Hz (mean = 7.2 Hz). To assess the influence of cut-off frequency, eight conditions were tested with the point of gaze being computed using eye rotation data filtered at cut-offs of 5, 6, 7, 8, 9, and 10 Hz (derived from the range of cut-off frequencies identified, see Figure 5 for an example), the autocorrelation frequency and the original untreated data. The accuracy MAE and precision RMSE-S2S measures were calculated between point of gaze and each of the floor targets (as above) for each of the data treatment conditions.

#### Gap Filling

A method utilized by Musial et al. (2011) was adapted to ascertain the impact of the data treatment processes. Raw data were imported into MATLAB where two matrices (origin position and gaze orientation) were created for each eye. The location of missing data points in these matrices was indexed. The gaze orientation data were processed to compute viewing angle (eye rotation) using the procedure outlined in the methods section (Figure 1, angle *a*). The origin position and viewing angle data were processed using a quintic spline and the auto-correlation function outlined in the methods section. The processed data were then used to compute the gaze vectors intercept of the floor plane using the previously detailed method. Using the index of missing data, any artificial data points were then removed and the floor intercept locations in the anterior–posterior direction for each eye were used to establish a baseline against which the effects of data treatment could be evaluated. That is to say, the original gaps were replaced to ensure artificial datapoints were omitted.

After establishing a baseline, akin to similar approaches taken in research evaluating the impact of data treatment on time series data (Musial et al., 2011), a second data set was created to represent missing data. To replicate missing data, a gap was created by removing values from the raw imported data. Using a custom MATLAB script, the position of the gap was designated using a random number generator function set to allocate a value ensuring a minimum threshold of 50 samples from the trial start and end points. The 50 sample cut-off was selected to strengthen the comparison by avoiding a situation where the created gap overlapped the data's start and end points (Grimshaw et al., 2019). The position of the removed samples was recorded. To examine the effect of gap size on the treated data, 11 gap size conditions were created with the size of the gap created being manipulated in five sample intervals between 0 (filtered signal data with no gap) to 50 samples (akin to 0–1 s of missing data). Previous research (Musial et al., 2011) undertaking similar comparisons have advocated large data samples to further strengthen analysis. As such, 50 randomly positioned gaps were created (non-simultaneously) within each eye's data set. This approach allowed a total of 1100 comparisons (11 participants, 2 eyes, 50 gaps) to be made for each of the gap size conditions. Following the creation of gaps, and data treatment process data either side of the gap MAE was calculated between the original and gap filled data to allow the effect of data treatment to be evaluated (Musial et al., 2011).

#### Statistical Analysis

For the MAE, RMS-S2S and STD measures, multilevel linear model repeated measures ANOVA's (alpha of 0.05) were undertaken using the nlme package in R (Pinheiro et al., 2020; RStudio Team, 2020). To explore the impact of target location, significant main effects were followed up using Tukey’s post-hoc tests, effect sizes (*r*) were calculated for these contrasts (Field et al., 2012). When comparing gap conditions, multilevel linear model repeated measures ANOVA's (alpha of 0.05) was undertaken using the nlme package in R (Pinheiro et al., 2020; RStudio Team, 2020). Normality was assessed via box and Q–Q plots with spurious outliers treated via winsorizing to 90% (Field et al., 2012; Ghosh & Vogt, 2012). To explore the impact of gap size, significant main effects were followed up using Tukey’s post-hoc tests, effect sizes (*r*) were calculated for these contrasts (Field et al., 2012).

## Results

The point of gaze floor intercept (Figure 4) indicated that all participants completed the task as requested. Visual inspection suggested that as look-ahead distance increased, point of gaze accuracy decreased. Several instances of data loss (e.g., at approximately 2.5 s, Figure 4) for one, or both, eyes were identified. Across all trials mean missing data equated to 187.95 ± 160.05 samples (*M* = 12.53% *SD* = 10.67%).

### Point of Gaze Accuracy

A multilevel repeated measures ANOVA indicated that floor target location had a significant effect on MAE, χ^2^(5) = 97.84, *p* < .001. Post-hoc Tukey contrasts revealed significant increases in MAE (all *p*s <.05) between targets at 1 m (*M* *=* 0.13 m) and targets at 4, 5, and 6 m (*M* *=* 0.33 m, *r* *=* .71; 0.56 m, *r* *=* .81; 0.82 m, *r* *=* .85, respectively); target at 2 m (*M* *=* 0.11 m) and targets at 4 m (*r* *=* .71), 5 m (*r* *=* .81) and 6 m (*r* *=* .86); target at 3 m (*M* *=* 0.19 m) and targets at 5 m (*r* *=* .74) and 6 m (*r* *=* .82); target at 4 m and targets at 5 m (*r* *=* .54) and 6 m (*r* *=* .74) targets at 5 and 6 m (*r* *=* .46); Figure 4).

**Figure 6.**
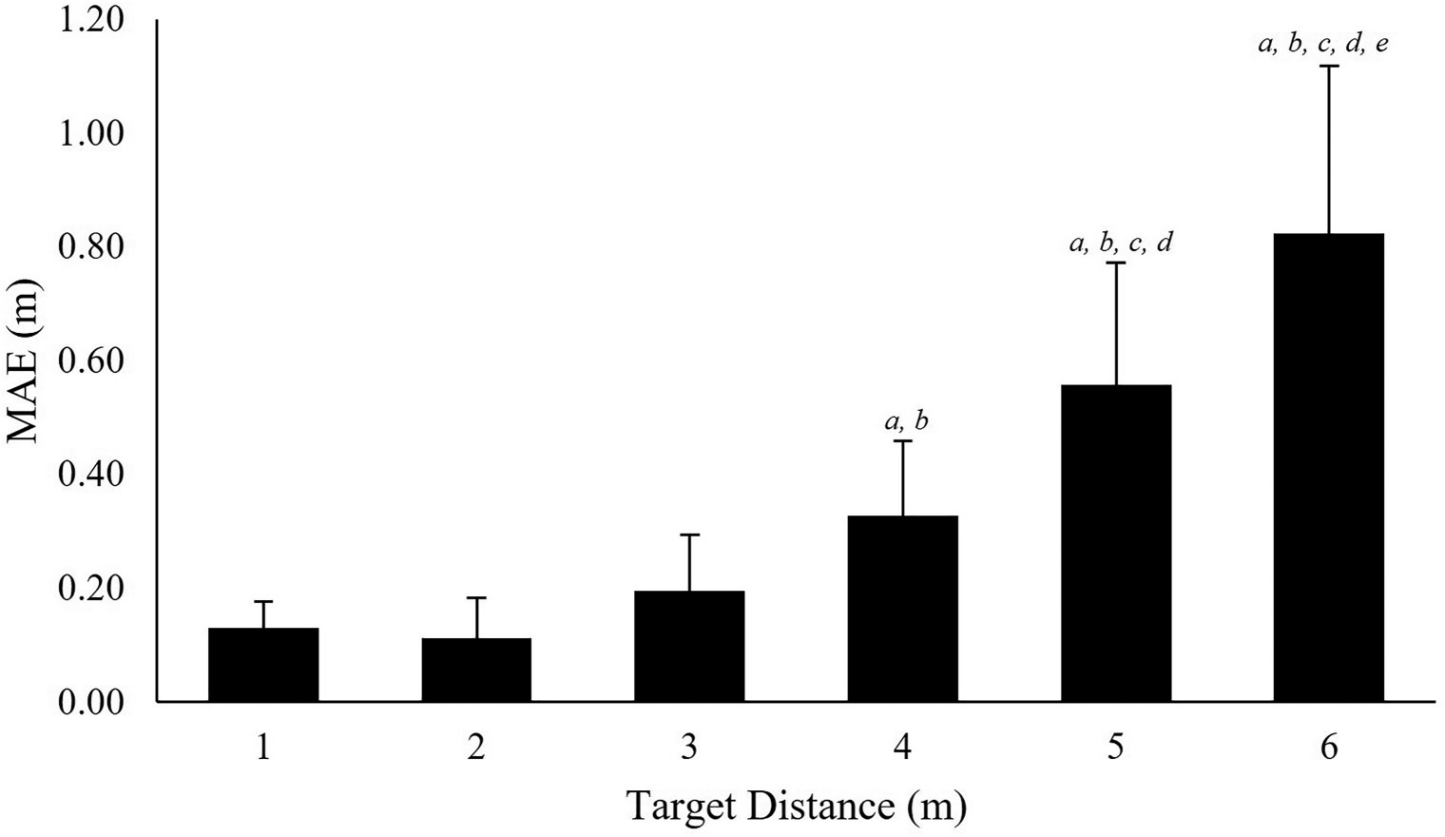
Accuracy at each floor-based target location. MAE is presented for each of the targets: *a* = significantly different (*p* < .05) to 1 m target; *b* = significantly different to 2 m target; *c* = significantly different to 3 m target; *d* = significantly different to 4 m target; *e* = significantly different to 5 m target. Error bars represent standard deviation.

The visual angle in degrees associated with the MAE for each floor-based target was computed. The results (Figure 6) indicated a mean accuracy of 2.55 ± 1.12° for all floor targets.

**Figure 7.**
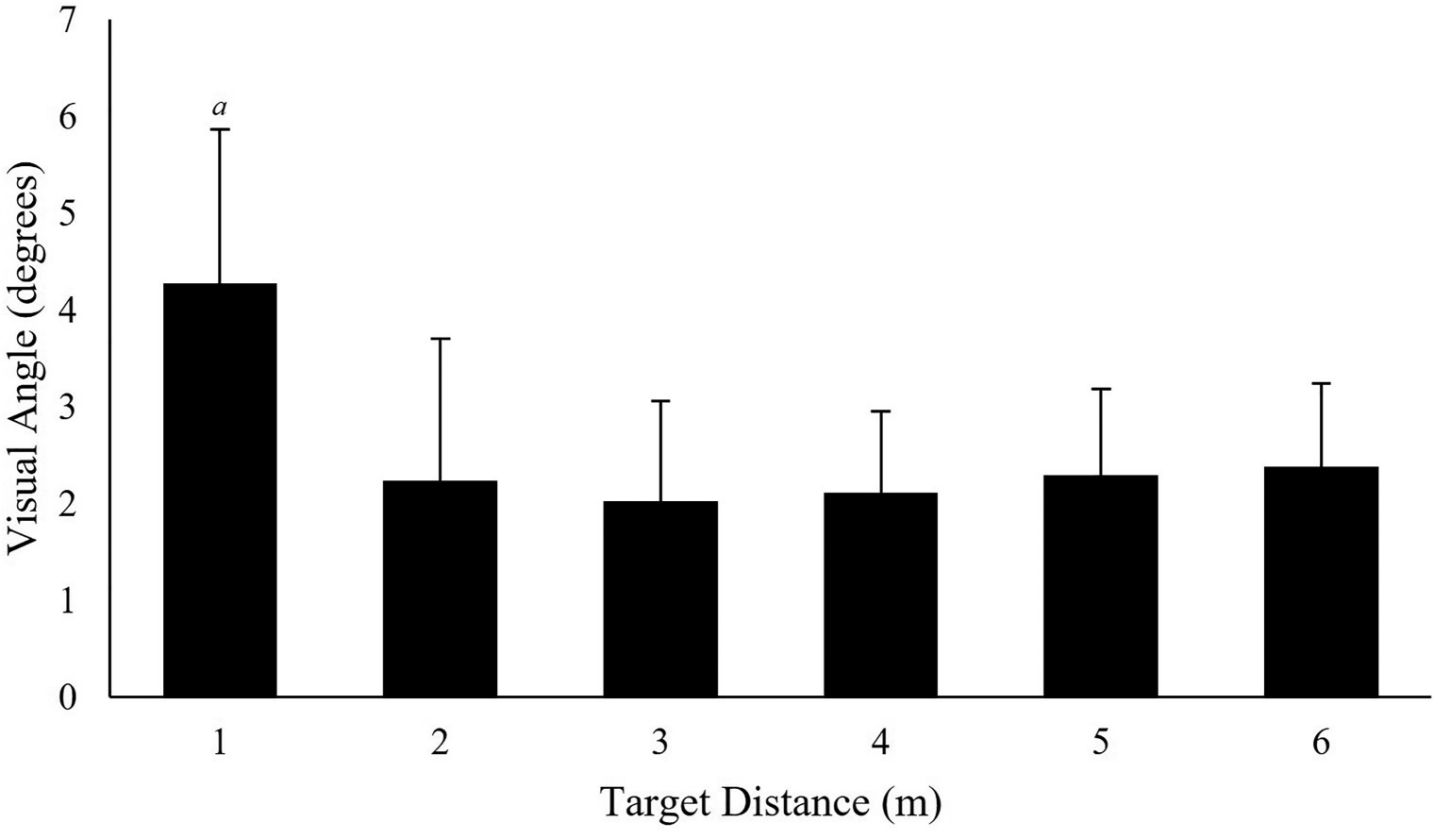
The visual angle associated with the MAE values for each floor-based target location. *a* = significantly different (*p* < .05) to 1 m target. Error bars represent standard deviation.

A multilevel repeated measures ANOVA indicated that floor target location had a significant effect on degrees of visual angle associated with the MAE, χ^2^(5) = 37.12, *p* <.001. Post-hoc Tukey contrasts revealed significant differences between target 1 and all other targets visual angle (*p* <.05, *r* *=* .55;.64;.65;.61;.59) for floor targets at 2 to 6 m, respectively) suggesting the most proximal target was associated with the largest inaccuracy (Figure 7). All other comparisons were non-significant (*p* >.05).

### Point of Gaze Precision

**Figure 8.**
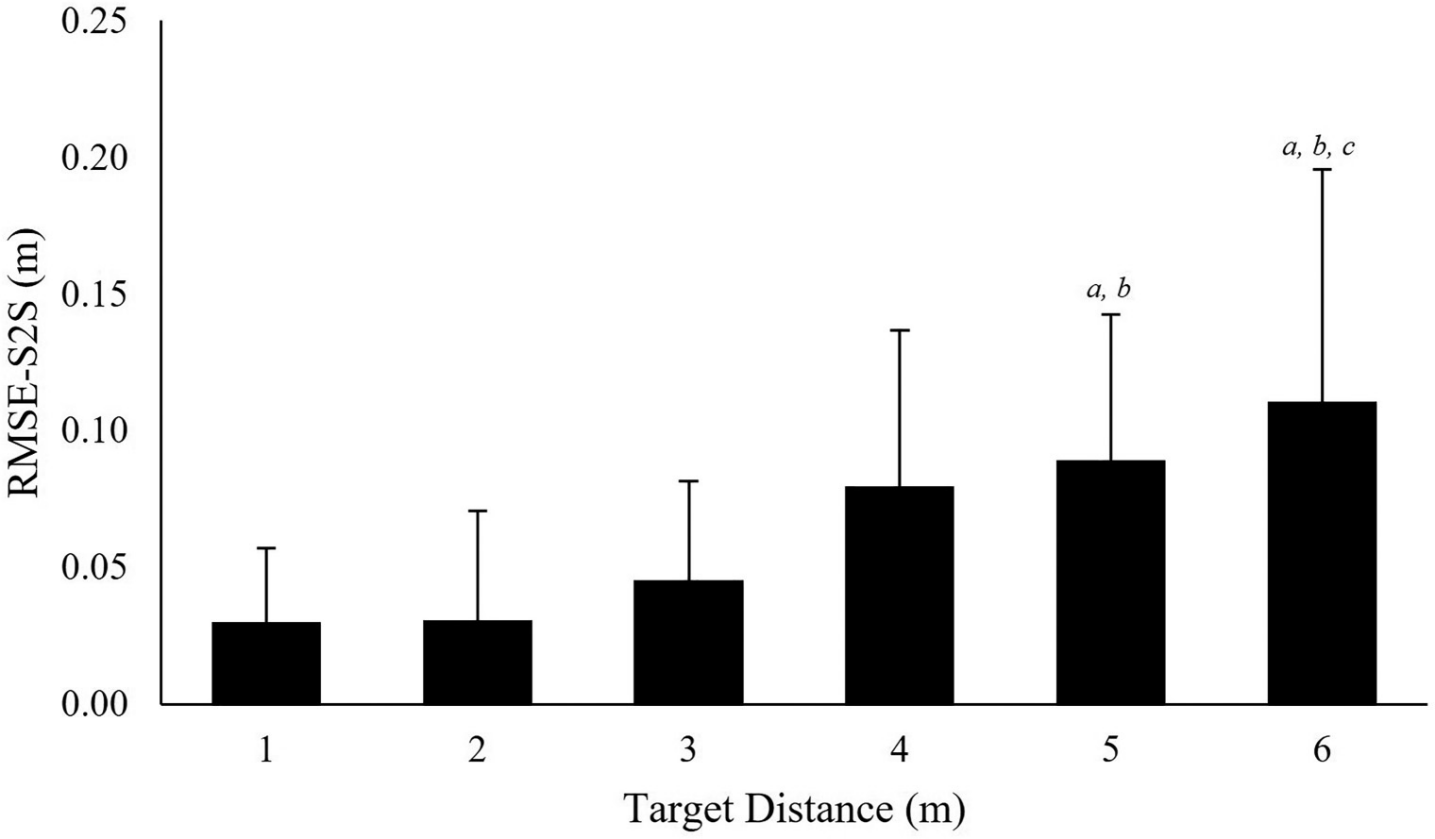
Precision at each floor-based target location. RMS-S2S is presented for each of the targets: *a* = significantly different (*p* < .05) to 1 m target; *b* = significantly different to 2 m target; *c* = significantly different to 3 m target. Error bars represent standard deviation.

**Figure 9.**
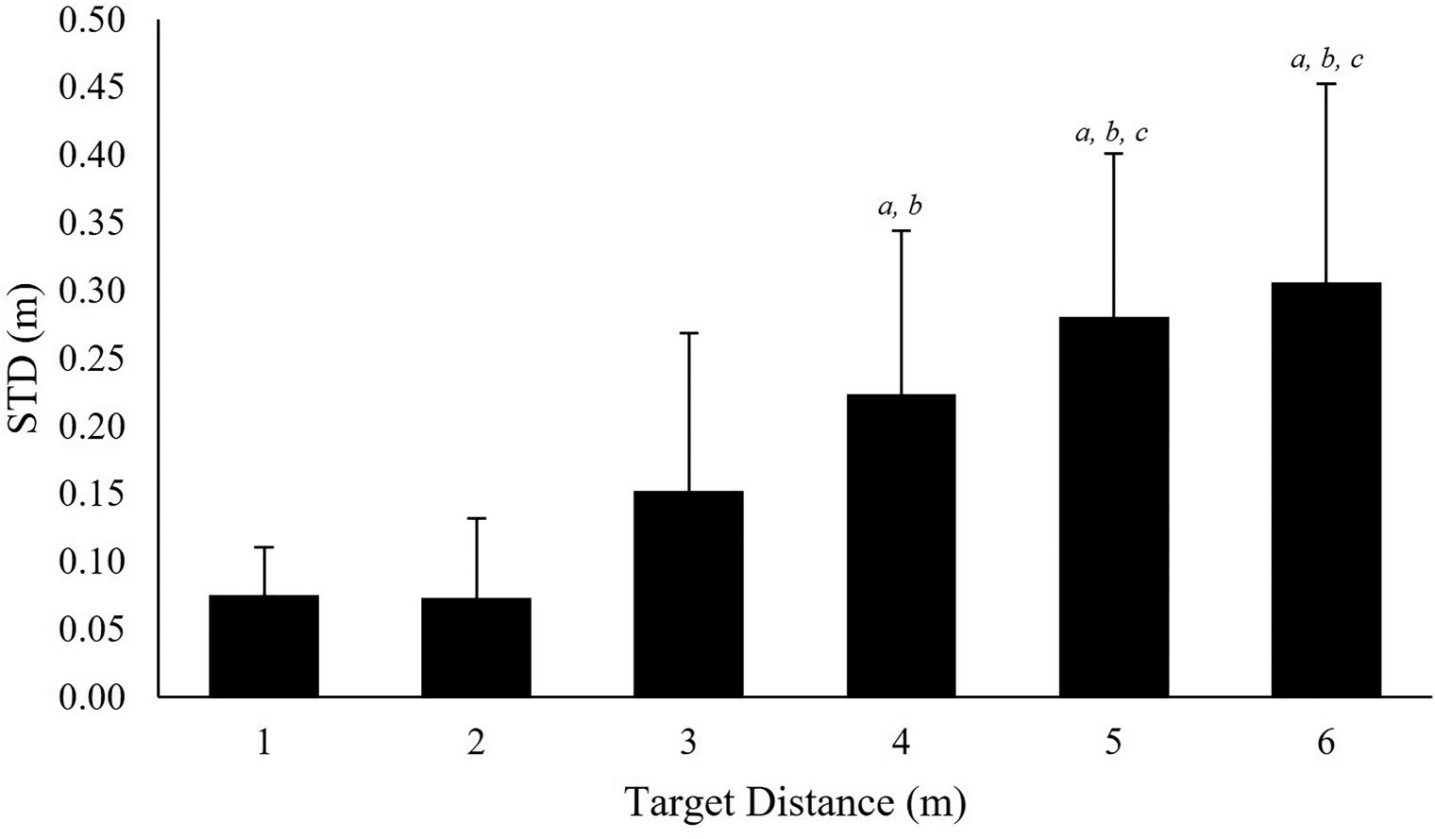
Precision at each floor-based target location. STD is presented for each of the targets: *a* = significantly different (*p* < .05) to 1 m target; *b* = significantly different to 2 m target; *c* = significantly different to 3 m target. Error bars represent standard deviation.

A multilevel repeated measures ANOVA indicated that floor target location had a significant effect on the RMSE-S2S precision measure, χ^2^(5) = 23.50, *p* <.001. Post-hoc Tukey contrasts revealed significant increases in RMSE-S2S (*p* <.05) between the target at 1 m (*M* *=* 0.03 m) and targets at 5 m (*M* *=* 0.09 m; *r* *=* .57), and 6 m (*M* *=* 0.11 m; *r* *=* .54); target at 2 m (*M* *=* 0.03 m) and targets at 5 m; (*r* *=* .53) and 6 m (*r* *=* .51); target at 3 m (*M* *=* 0.05 m) and target at 6 m (*r* *=* .45; Figure 8). Moreover, a multilevel repeated measures ANOVA on the STD precision measure indicated that target distance had a significant effect, χ^2^(5) = 51.14, *p* < .001. Post-hoc Tukey contrasts revealed significant increases in STD (*p* <.05) between target at 1 m (*M* *=* 0.08 m) and targets at 4, 5, and 6 m (*M* *=* 0.22 m, *r* *=* .64; 0.28 m, *r* *=* .76; 0.31 m, *r* *=* .73, respectively); target at 2 m (*M* *=* 0.07 m) and targets at 4 m (*r* *=* .62), 5 m (*r* *=* .74), and 6 m (*r* *=* . 72); target at 3 m (*M* *=* 0.15 m) and targets at 5 m (*r* *=* .48) and 6 m (*r* *=* .50; Figure 9).

### Signal Processing

Separate one-way ANOVAs (RStudio, 2020), identified no significant main effect for treatment condition for measures of MAE, *F*(7,520) = 0.003, *p* = 1.000, η2 = 0.013 or RMSE-S2S, *F*(7,520) = 0.969, *p* = 0.453, η2 = 0.013 (Table 1).

**Table 1.** Accuracy and Precision of Different Data Filter Cut-off Frequencies.

	MAE (m)		RMSE-S2S (m)	
	Mean	SD	Mean	SD
Untreated data	0.356	0.038	0.064	0.060
Autocorrelation	0.363	0.037	0.052	0.042
5 Hz	0.359	0.037	0.047	0.037
6 Hz	0.360	0.037	0.050	0.040
7 Hz	0.360	0.037	0.053	0.044
8 Hz	0.360	0.037	0.056	0.046
9 Hz	0.361	0.037	0.058	0.049
10 Hz	0.362	0.037	0.061	0.051

### Gap Filling

**Figure 10.**
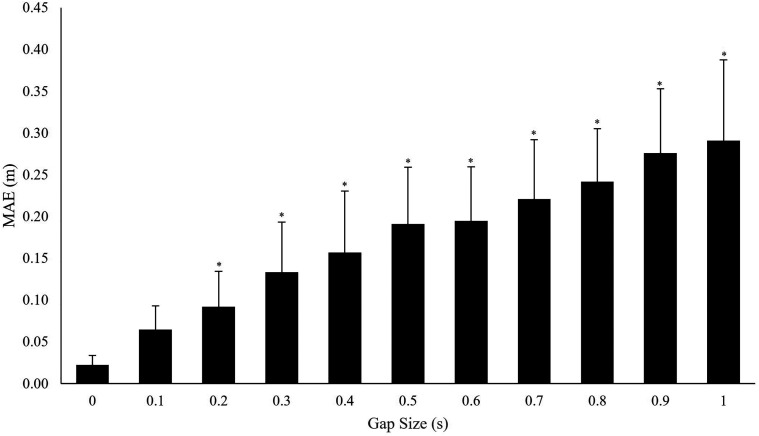
MAE associated with treating different gap sizes. 0 gap size represents comparison between processed and unprocessed data. * = significantly different (*p* < .05) to processed data with no gaps. Error bars represent standard deviation.

The results comparing the MAE between treated data and data with created gaps (Figure 10) showed that gap size had a significant effect on MAE, χ^2^(11) = 215.16, *p* < .001. Post-hoc Tukey contrasts revealed no significant difference between processed data with no created gaps (0 s gap size) and unprocessed data (*p* = .150, *r* = .15). Significant differences (all *p*s <.05) were identified in MAE between treated data with gaps of 0 samples and data treating gap sizes of 0.1 s (10 samples) or greater (*r* *=* .74;.79;.78;.85;.87;.88;.92;.91;.88 for gap sizes of 0.1 to 1 s, respectively).

## Discussion

Integrating mobile eye tracking and optoelectronic motion capture presents a method of translating gaze data to a world centered frame of reference (e.g., the laboratory co-ordinate system). Such an integration has provided an additional method of collecting and analyzing human perceptual-motor behaviors. Our primary aim was to assess the accuracy and precision of eye tracking data collected using the integrated Tobii Pro Glasses 2 and Qualisys motion capture system. Gaze data accuracy and precision were considered as participants were asked to attend to floor-based targets at distances between 1 and 6 m. Consistent with geometric necessity results indicated a reduction in accuracy and precision associated with increased look-ahead distances. However, inconsistent with a geometric approach, reduced accuracy (e.g., increased error) was acknowledged at the most proximal floor target. The second aim was to explore if signal processing techniques may help overcome limitations, such as data loss and decreased accuracy and precision in eye tracking data with increased viewing distance and angle. Results indicated that data treatment did not significantly alter data quality measures. Most notably, non-significant differences in accuracy measures when interpolating gaps of up to 0.1 s suggest that gap filling may present a solution to small gaps (e.g., less than 0.1 s) occurring within gaze data sets. This result is consistent with gap filling thresholds outlined applied in extant gaze research (Hessels et al., 2017). These findings and their implications shall be explored in more detail in the subsequent sections.

### Accuracy and Precision of Gaze Data

MAE results showed that point of gaze accuracy, in relation to world space, decreased at greater look-ahead distances (Figure 6). Specifically, when looking at Target 1 MAE was 0.13 m whereas when looking at target 6 MAE was a significantly greater 0.82 m. The reduced accuracy at greater viewing distances may be particularly important throughout perceptual-motor control research, such as studies of human locomotion (Ellmers et al., 2016; Hessels, Benjamins et al., 2020) and dynamic sport actions including goalkeeping in the penalty kick (Navia et al., 2017), where eye movements are characterized by fixations on floor-based locations. To further explore the effect of viewing distance on accuracy, the visual angle associated with each of the floor-based target locations MAE was computed (Figure 7). Our results suggested that accuracy, in degrees, at the most proximal target (4.27°) was significantly less accurate than all other targets. This reduction in accuracy may be associated with the eye tracker's capability to accurately detect pupil location at larger viewing angles (Hornof & Halverson, 2002; Pastel et al., 2020) or because gaze is further from the glasses (central) calibration point (Niehorster, Santini et al., 2020) as would be caused by floor-based proximal locations. In support of this suggestion, comparing our findings to research examining eye movements oriented towards wall mounted targets, MacInnes et al. (2018) reported a mean accuracy of 1.42 ± 0.58° for fixations on eye-height targets at distances up to 3 m (0.8°, 1.6°, and 1.8° for 1–3 m, respectively). Comparatively, we reported a mean accuracy of 2.85 ± 1.24° for floor-based targets up to the same distance (4.27°, 2.24°, and 2.02° for 1–3 m, respectively; Figure 7). The reduced accuracy presented in the current study affirms that the requirement to make proximal floor-based visual fixations appears to negatively influence accuracy. Considering the MAE and visual angle data together, the increased MAE at viewing distances of 4 m and greater (Figure 6) and larger viewing angles associated with more proximal locations (Figure 7) aligns with our hypothesis suggesting that both larger viewing angles (e.g., proximal locations) or large viewing distances (e.g., distal locations) may invite accuracy errors (MacInnes et al., 2018). In addition to distance related inaccuracy, research has shown that eye tracker slippage (device movement relative to the participants head) caused by participant movement, speaking, or facial expressions can reduce data quality (Niehorster, Santini et al., 2020). Although Niehorster, Santini et al. (2020) found Tobii Pro Glasses 2 to be fairly robust to slippage induced inaccuracy, the link between larger viewing distances and greater MAE emphasizes that researchers must take greater care to reduce all potential sources of error when collecting gaze data from participants attending to distal locations.^7^

Results identified that precision measures (RMSE-S2S and STD) increased at greater look-ahead distances (Figures 8 and 9). Specifically, precision measures at target 1 were 0.03 m RMSE-S2S and 0.08 m STD whereas target 6 measures were 0.11 m RMSE-S2S and 0.31 m STD. The reduced precession observed at greater viewing distances has potential consequences for future eye movement research. For example, automated gaze behavior classification functions (e.g., fixation classification filters) are a common feature of eye tracking analysis packages. Within these packages, fixations are typically classified relative to a head-based frame of reference as periods of stable eye movement (in degrees) (Hessels et al., 2018; Kothari et al., 2020). However, automatic gaze classification functions are limited when applied to free-moving tasks, such as ambulation, due to the capacity of participants to adjust eye position to counteract head movement (Hessels et al., 2018). Alternatively, adaptive locomotion literature has commonly classified fixations as periods of gaze stability in relation to areas of interest in the world (e.g., Ellmers et al., 2020). Integration of eye tracking with optoelectronic motion capture offers the capacity to express point of gaze within the environment thereby affording event classification during free-moving tasks. However, the increased variability (e.g., STD, Figure 9) identified when participants attended to distal (>3 m) floor-based targets may indicate a possible challenge to automating such an approach. Specifically, the geometry of the experimental set up used in the current study appeared to introduce a nonlinear increase in noise as a function of distance. Whilst utilizing the gaze orientation signal for event classification may negate the issue of nonlinear noise with increasing distance (e.g., Hessels et al., 2017; Nyström & Holmqvist, 2010), it would not provide a possible solution to determining gaze events within dynamic everyday environments that require changes in look-ahead distance to, for example, safely plan routes through cluttered environments (e.g., Matthis et al., 2018) or intercept a distal foot target (Hildebrandt & Cañal-Bruland, 2020).

Despite automated event classification becoming widespread throughout gaze behavior research, the algorithms that facilitate behavior classification are dependent on a head-based frame of reference and are often contained within software packages that offer researchers limited flexibility over the analysis process (Niehorster, Hessels et al., 2020; Vansteenkiste et al., 2014). Previous work reviewing the treatment of gaze data has acknowledged that researchers should be mindful of the fact that results may be heavily dependent on the algorithms and parametrizations used (Kiefer, et al., 2017). Our findings reinforce this point and signpost that further work is required to investigate the ramifications of viewing distance on automatic gaze behavior classification when head to world transformation of gaze data is required, particularly regarding dynamic tasks where floor-based viewing distances exceed 3 m.

The reduction in data quality associated with increased viewing distances appears to be consistent for both the integrated data collection methods examined in the current study and scene camera data collection methods described in previous research (e.g., MacInnes et al., 2018). As such, selecting a viewing distance upper limit prior to the commencement of an experiment may offer a means of promoting data quality. Specifically, given that there were no differences between accuracy (MAE) in precision (STD) measures when participants fixated on targets between 1 and 3 m away, a cut-off distance of 3 m may be considered appropriate for research measuring floor-based point of gaze. Alternatively, viewing distance-based thresholds may be quantified relative to specific research questions. For example, research examining locomotor pointing in the context of a long jump task have considered areas of interest of 20 × 120 cm (*x* and *y* dimensions) and approach distances of between 15.75 and 24.69 m (Hildebrandt & Cañal-Bruland, 2020). At such distances, reduced accuracy may hinder the ability to accurately establish point of gaze within a relatively small area of interest. Given the variable (Dicks et al., 2017) and environment specific nature of gaze behaviors, and the variation in quality reported between participants (Blignaut & Wium, 2014; Thibeault, 2019), future research may wish to further consider gaze data quality to establish a viewing distance threshold that informs decisions regarding gaze data analysis relevant to individual study design. Although adopting a distal cut-off for point of gaze data may help account for these limitations in future work, further research is clearly warranted.

### Processing Gaze Data

Results from the current study highlight less precise data as occurring at larger viewing distances and viewing angles (see Figure 4 for an example). Instances of data loss were also noted (*M* = 12.53% *SD* = 10.67%) which corresponded to instances of either retroreflective marker or pupil occlusion which resulted in failure to create the origin eye position or measure gaze orientation. Consistent with this observation, previous research has separately identified several limitations associated with data collected using optoelectronic motion capture or eye tracking, with limitations including noise and missing data (Camargo et al., 2020; Hessels et al., 2017; Kurzhals et al., 2017; Spörri et al., 2016). Collecting data through the integrated system offers researchers scope to apply signal processing techniques (Grimshaw et al., 2019).

#### Filtering Gaze Data

Findings from the current study revealed that compared to the untreated point of gaze data, applying a 4th-order Butterworth filter at the autocorrelation determined cut-off frequency did not significantly change gaze data quality measures. Furthermore, no changes were identified when comparing untreated data to data filtered using a standardized cut-off frequency. These findings also suggest that using the autocorrelation function to identify a cut-off frequency does not significantly change data quality compared to filtering using a single cut-off frequency. However, perceptual-motor control literature has frequently acknowledged that gaze behaviors are influenced by a myriad of factors including the performance environment (Matthis et al., 2018), the task being performed (Huys & Beek, 2010), and the participant's abilities (Ellmers et al., 2020). Accordingly, the use of the autocorrelation function may present a flexible method of treating gaze data that respects the diversity of perceptual-motor behaviors not captured within the data set of the current study. For instance, further research is required to establish the effect of these data treatment processes on gaze data collected in settings such as fast-paced movement or target interception (e.g., Navia et al., 2017). Furthermore, based on the varied data qualities present within each trials (e.g., reduced precision at greater look-ahead distances; Figure 4), future research may wish to consider if autocorrelation approaches could be used to establish adaptive filter parameters which account for changes in the signals frequency content (Crenna et al., 2021; Erer, 2007).

#### Gap Filling Gaze Data

Previous literature pertaining to data collected via both optoelectronic capture and eye tracking have highlighted instances of missing data (Duchowski, 2007; Hessels et al., 2015; Spörri et al., 2016). Our findings were consistent, noting that missing data was commonly overserved. To overcome this limitation, a quintic spline was applied to the data set. Because reduced accuracy has been identified as a limitation of treating larger gaps using splines (Howarth & Callaghan, 2010) our aim was to compare treated and original data when filling gaps of different sizes (Musial et al., 2011).

When not filling gaps, no significant difference was identified between treated and the original gaze intercept data. The absence of differences indicates that the treatment process did not significantly change point of gaze location when there were no gaps in the data. Further, the lack of significant difference up to a gap size of 0.1 s (Figure 10) suggests that the data treatment process was sufficient to overcome missing data spanning less than 0.1 s. However, significant differences were observed at gap sizes of 0.2 s or more, which is consistent with previous research suggesting increased error when treating larger gaps (Howarth & Callaghan, 2010) and gap sizes thresholds (0.1 s) outlined previously in gaze methodology research (Hessels et al., 2017). To account for this, establishing a maximum gap size threshold based on the largest non-significant gap size may present one method of limiting the accuracy reduction associated with treating gaze data. For example, our findings suggest that the data treatment method was sufficient to overcome gaps in the data up to an upper gap size threshold of 0.1 s. Although this upper gap size threshold may offer a guide for future research, caution is recommended in universally applying this threshold. Accordingly, future research may undertake a similar analysis in order to consider the implications associated with treating data collected across a wider array of research methods. Alternatively, considering MAE associated with each gap size may present a method of establishing an upper gap size threshold. For example, the MAE associated with filling gaps of 30 samples (0.19 m) is similar to the MAE associated with look-ahead distances of 3 m (0.19 m). As such, it may be appropriate for researchers to establish a threshold based on the size, or location, of areas of interest being evaluated, with larger areas being less sensitive to the accuracy loss incurred by filling larger gaps.

Although similar procedures are offered through commercial software packages (e.g. Tobii pro lab), the algorithms in such software have been considered inflexible, with results being influenced by a process that researchers have limited scope to control (Niehorster, Hessels, et al., 2020). Moreover, such packages often focus on classifying gaze behaviors rather than promoting measurement accuracy (Hessels et al., 2017; Kiefer et al., 2017). Considering that these processes influence the reproducibility of eye tracking research, investigators have recommended, and developed, “open source” eye tracking analysis packages (Niehorster, Hessels et al., 2020). Akin to these approaches, exporting the eye position (motion capture) and orientation (eye tracker) data directly into processing software (e.g., MATLAB) vastly increases the range of treatment options available. Moreover, the ability to analyze gaze data using “open source” or processing software invites future researchers to consider the benefits of evaluating different (e.g., left eye, right eye, version, or vergence) signals expressed with a world reference frame (Hooge et al., 2019). Furthermore, greater focus on the development of algorithms for the categorization of gaze events, such as fixations, pursuits, and saccades, while the head is free (Kothari et al., 2020; Niehorster, Hessels, et al., 2020) could be beneficial to the wider research community. Future research may wish to consider if spatiotemporal analysis of world-space gaze location may offer a parsimonious approach to categorizing gaze events. Overall, this represents a major advantage to gaze behavior research and signifies that the integration may offer a platform that encourages greater clarity and innovation regarding the capture and analysis of gaze data.

### Conclusion

The primary aim of this study was to assess the accuracy and precision of eye tracking data collected using the integrated Tobii Pro Glasses 2 and Qualisys motion capture system. Gaze data accuracy and precision were evaluated as participants attend to floor-based targets between distances of 1–6 m. Supporting our primary hypothesis, which predicted reduced accuracy when participants attend the most proximal and distal floor targets, we found that both accuracy and precision were reduced at viewing distances of greater than 3 m. Further, we found evidence suggesting that greater viewing angles, such as those caused by attending to ground-based locations at approximately 1 m, also reduced accuracy. These findings highlight a need for future research to review the quality of gaze data collected at distances greater than 3 m. As the increased error at greater viewing distances can be defined geometrically and is applicable across a wide range of contexts, future researchers should consider the implications of data quality throughout experimental design particularly when utilizing a combination of eye tracking and motion capture. With this in mind, future research may wish to collect gaze data with and without motion capture integration to compare the quality of data collected through both methods.

The second aim was to explore if decreased accuracy and precision of gaze data with increased viewing distance and angle, as well as loss of gaze data could be addressed via signal processing methods. We found that filtering the data using a low-pass Butterworth filter at a variety of cut-off frequencies identified using an autocorrelation function did not significantly change data quality, however, gap-filling using a quintic spline was sufficient to overcome missing data spanning less than 0.1 s. Future research should build on these findings and consider if procedures outside of the scope of this paper, such as adaptive filtering, offer solutions to these issues. Finally, with the integration of gaze and motion capture becoming a viable methodology in the study of human behavior, we have provided several primary considerations for the collection and analysis of gaze data that may help inform future methodological decisions.

## Supplemental Material

sj-zip-1-ipe-10.1177_20416695221116652 - Supplemental material for Evaluating the integration of eye-tracking and motion capture technologies: Quantifying the accuracy and precision of gaze measures Click here for additional data file. Supplemental material, sj-zip-1-ipe-10.1177_20416695221116652 for Evaluating the integration of eye-tracking and motion capture technologies: Quantifying the accuracy and precision of gaze measures by Rhys Hunt, Tim Blackmore, Chris Mills, and Matt Dicks in i-Perception

## Notes

1. Please refer to: https://www.qualisys.com/webinars/tobii-pro-eye-tracking-and-motion-capture-integration.

2. For full detailed method, see Matthis et al. (2018).

3. Normally the point of gaze is overlaid onto footage captured through a forward-facing “scene” camera mounted on the glasses frame, which allows for gaze location examination to be undertaken via frame-by-frame analysis or via a variety of behaviour identifying filters provided by the Tobii Pro Lab analysis software.

4. This method can be expanded to allow computation of the *y* (medial-lateral) axis floor intercept, which enables a computation of a set of coordinates detailing the *z* axis intercept for non-linear tasks. 

5. The methodological decision to exclude data has been based on research that uses a scleral coil to measure eye movement velocity, however, differences between scleral coil and pupil-based measurement systems have been identified (Hooge et al., 2016). We therefore advise future researcher may need to develop appropriate cut-off methods based on mobile eye tracking systems.

6. For precision measures with a head-based reference frame, see online Supplemental material.

7. Niehorster et al., (2020) suggest researchers may identify slippage induced error by validating gaze position at the end of a period of data collection.

## References

[bibr1-20416695221116652] Adler D. (1999). Metric handbook planning and design data (2nd ed.). Architectural Press.

[bibr2-20416695221116652] Benjamins J. Hessels R. Hooge I . (2018). Gazecode: Open-source software for manual mapping of mobile eye-tracking data. In *ETRA '18: 2018 Symposium on Eye Tracking Research and Applications*, June 14–17, 2018, Warsaw, Poland (p. 4). New York, NY, USA: ACM. 10.1145/3204493.3204568

[bibr3-20416695221116652] Blignaut P. Wium D . (2014). Eye-tracking data quality as affected by ethnicity and experimental design. Behavior Research Methods, 46(1), 67–80. 10.3758/s13428-013-0343-0 23609415

[bibr4-20416695221116652] Burger B. Puupponen A. Jantunen T . (2018). Synchronizing eye tracking and optical motion capture: How to bring them together. Journal of Eye Movement Research, 11(2), 1–16. 10.16910/jemr.11.2.5 PMC7733527 33828688

[bibr5-20416695221116652] Camargo J. Ramanathan A. Csomay-shanklin N . (2020). Automated gap-filling for marker-based biomechanical motion capture data. Computer Methods in Biomechanics and Biomedical Engineering, 0(0), 1–10. 10.1080/10255842.2020.1789971 32654510

[bibr6-20416695221116652] Chai T. Draxler R. R . (2014). Root mean square error (RMSE) or mean absolute error (MAE)? -Arguments against avoiding RMSE in the literature. Geoscientific Model Development, 7(3), 1247–1250. 10.5194/gmd-7-1247-2014

[bibr7-20416695221116652] Challis J .. (1999). A procedure for the automatic determination of filter cutoff frequency for the processing of biomechanical data. Journal of Applied Biomechanics, 15(3), 303–317. 10.1123/jab.15.3.303

[bibr8-20416695221116652] Cohen J. (1988). Statistical power analysis for the behavioural sciences. Laurance Erlbaum Associates.

[bibr9-20416695221116652] Collewijn H. Erkelens C. J. Steinman R. M . (1988). Binocular co-ordination of human vertical saccadic eye movements. The Journal of Physiology, 404(1), 183–197. 10.1113/jphysiol.1988.sp017285 3253430 PMC1190821

[bibr10-20416695221116652] Cornus S. Laurent M. Laborie S . (2009). Perception-movement coupling in the regulation of step lengths when approaching an obstacle. Ecological Psychology, 21(4), 334–367. 10.1080/10407410903320991

[bibr11-20416695221116652] Crenna F. Rossi G. B. Berardengo M . (2021). Filtering biomechanical signals in movement analysis. Sensors, *21*(13), 1–17. 10.3390/s21134580. PMC8271607 34283131

[bibr12-20416695221116652] Davis D. J. Challis J. H . (2020). Automatic segment filtering procedure for processing non-stationary signals. Journal of Biomechanics, 101, 109619. 10.1016/j.jbiomech.2020.109619 31952818

[bibr13-20416695221116652] Dicks M. Button C. Davids K. Chow Y. J. van der Kamp J . (2017). Keeping an eye on noisy movements: On different approaches to perceptual-motor skill research and training. Sports Medicine, 47(4), 575–581. 10.1007/s40279-016-0600-3 27497599

[bibr14-20416695221116652] Dicks M. Davids K. Button C . (2010). Individual differences in the visual control of intercepting a penalty kick in association football. Human Movement Science, 29(3), 401–411. 10.1016/j.humov.2010.02.008 20359763

[bibr15-20416695221116652] Dietzsch M. David S. Dupré T. Komnik I. Potthast W . (2017). Comparing estimated and measured muscle activation during highly dynamic and multidirectional movements-a validation study. ISBS Proceedings Archive, 35(1), 18.

[bibr16-20416695221116652] Domínguez-Zamora J. F ., Gunn S. M ., & Marigold D. S . (2018). Adaptive gaze strategies to reduce environmental uncertainty during a sequential visuomotor behaviour. Scientific Reports, 8(1), 1–13. 10.1038/s41598-018-32504-0 30237587 PMC6148321

[bibr17-20416695221116652] Domínguez-Zamora J. F. Marigold D. S . (2021). Motives driving gaze and walking decisions. Current Biology, 31, 1–11. 10.1016/j.cub.2021.01.069 33600769

[bibr18-20416695221116652] Draschkow D. Võ M. L. H . (2016). Of “what” and “where” in a natural search task: active object handling supports object location memory beyond the object’s identity. Attention, Perception, and Psychophysics, 78(6), 1574–1584. 10.3758/s13414-016-1111-x 27165170

[bibr19-20416695221116652] Duchowski A. Medlin E. Cournia N. Murphy H. Gramopadhye A. Nair S. Vorah J. Melloy B . (2002). 3-D Eye movement analysis. Behavior Research Methods, Instruments, and Computers, 34(4), 573–591. 10.3758/BF03195486 12564561

[bibr20-20416695221116652] Duchowski A. T. (2007). *Eye Tracking Methodology* . 10.1007/978-3-319-57883-5

[bibr21-20416695221116652] Ellmers T. J. Cocks A. J. Doumas M. Williams A. M. Young W. R . (2016). Gazing into thin air: The dual-task costs of movement planning and execution during adaptive gait. PLoS ONE, 11(11), 1–20. 10.1371/journal.pone.0166063 PMC5100909 27824937

[bibr22-20416695221116652] Ellmers T. J. Cocks A. J. Young W. R . (2020). Evidence of a link between fall-related anxiety and high-risk patterns of visual search in older adults during adaptive locomotion. Journals of Gerontology - Series A Biological Sciences and Medical Sciences, 75(5), 961–967. 10.1093/gerona/glz176 31362302 PMC7164535

[bibr23-20416695221116652] Erer K. S . (2007). Adaptive usage of the Butterworth digital filter. Journal of Biomechanics, 40(13), 2934–2943. 10.1016/j.jbiomech.2007.02.019 17442321

[bibr24-20416695221116652] Essig K. Prinzhorn D. Maycock J. Dornbusch D. Ritter H. Schack T . (2012). Automatic analysis of 3D gaze coordinates on scene objects using data from eye-tracking and motion-capture systems. Association for Computing Machinery, 1(212), 37–44.

[bibr25-20416695221116652] Faul F. Erdfelder E. Lang A. G. Buchner A . (2007). G*Power 3: A flexible statistical power analysis program for the social, behavioral, and biomedical sciences. Behaviour Research Methods, 2(39), 175–191. 10.3758/BF03193146 17695343

[bibr26-20416695221116652] Field A. Miles J. Field Z. (2012). Discovering statistics using R. Sage.

[bibr27-20416695221116652] Frank M. C. Vul E. Johnson S. P. (2009). Development of infants’ attention to faces during the first year. Cognition, *110*(2), 160–170. 10.1016/j.cognition.2008.11.010 PMC2663531 19114280

[bibr28-20416695221116652] Ghosh D. Vogt A . (2012, July). Outliers: An evaluation of methodologies. In Joint statistical meetings (Vol. 2012).

[bibr29-20416695221116652] Grimshaw P. Cole M. Burden A. Fowler N. (2019). Instant notes in sport and exercise biomechanics (2nd ed.). Routledge.

[bibr30-20416695221116652] Hessels R. S. Andersson R. Hooge I. Nystrom M. Kemner C . (2015). Consequences of eye color, positioning, and head movement for eye-tracking data quality in infant research. Infancy, 20(6), 601–633. 10.1111/infa.12093

[bibr31-20416695221116652] Hessels R. S. Benjamins J. S. van Doorn A. J. Koenderink J. J. Holleman G. A. Hooge I. T. C . (2020). Looking behavior and potential human interactions during locomotion. Journal of Vision, 20(10), 5. 10.1167/jov.20.10.5 PMC7545070 33007079

[bibr32-20416695221116652] Hessels R. S. Niehorster D. C. Holleman G. A. Benjamins J. S. Hooge I. T. C. (2020). Wearable technology for “real-world research”: realistic or not? Perception, *49*(6), 611–615. 10.1177/0301006620928324 PMC7307000 32552490

[bibr33-20416695221116652] Hessels R. S. Niehorster D. C. Kemner C. Hooge I. T. C . (2017). Noise-robust fixation detection in eye movement data: Identification by two-means clustering (I2MC). Behavior Research Methods, 49(5), 1802–1823. 10.3758/s13428-016-0822-1 27800582 PMC5628191

[bibr34-20416695221116652] Hessels R. S. Niehorster D. C. Nyström M. Andersson R. Hooge I. T. C. (2018). Is the eye-movement field confused about fixations and saccades? A survey among 124 researchers. Royal society open science, *5*(8), 1–23. 10.1098/rsos.180502 PMC6124022 30225041

[bibr35-20416695221116652] Higuchi T . (2013). Visuomotor control of human adaptive locomotion: Understanding the anticipatory nature. Frontiers in Psychology, 4(277), 1–9. 10.3389/fpsyg.2013.00277 23720647 PMC3655271

[bibr36-20416695221116652] Hildebrandt A. Cañal-Bruland R . (2020). Is gait-based visual regulation identical to gaze-based visual regulation in inexperienced athletes’ long jump run-ups? Human Movement Science, 73(September), 102681. 10.1016/j.humov.2020.102681 32942208

[bibr37-20416695221116652] Holmqvist K. Nyström M. Andersson R. Dewhurst R. Jarodzka H. Weijer J. van de . (2010). Eye tracking A comprehensive guide to methods and measures (Vol. 53, Issue 9). Oxford University Press.

[bibr38-20416695221116652] Hooge I. Holmqvist K. Nyström M . (2016). The pupil is faster than the corneal reflection (CR): are video based pupil-CR eye trackers suitable for studying detailed dynamics of eye movements ? Vision Research, 128, 6–18. 10.1016/j.visres.2016.09.002 27656785

[bibr1039-20416695221116652] Hooge, I. T., Holleman, G., Haukes, M., & Hessles, R. (2019). Gaze tracking in humans: One eye is sometimes better than two. *Behaviour Research Methods*, *51*, 2712–2721. 10.1016/B978-0-323-60984-5.00062-7 PMC6877490 30350022

[bibr39-20416695221116652] Hornof A. J. Halverson T . (2002). Cleaning up systematic error in eye-tracking data by using required fixation locations. Behavior Research Methods, Instruments, and Computers, 34(4), 592–604. 10.3758/BF03195487 12564562

[bibr40-20416695221116652] Houpt J. W. Frame M. E. Blaha L. M. (2018). Unsupervised parsing of gaze data with a beta-process vector auto-regressive hidden markov model. In Behavior research methods, *50*(5), 2074–2096. 10.3758/s13428-017-0974-7 29076106

[bibr41-20416695221116652] Howarth S. Callaghan J . (2010). Quantitative assessment of the accuracy for three interpolation techniques in kinematic analysis of human movement Computer Methods in Biomechanics and Biomedical Engineering, *13*(6), 847–855. 10.1080/10255841003664701 21153975

[bibr42-20416695221116652] Huys R. Beek P. J . (2010). The coupling between point-of-gaze and ballmovements in three-ball cascade juggling: The effects of expertise, pattern and tempo. The coupling between point-of-gaze and ball movements in three-ball cascade juggling: The eþ ects of expertise, pattern an. Journal of Sports Sciences, 20(3), 171–186. 10.1080/026404102317284745 11999474

[bibr43-20416695221116652] Jantunen T. Puupponen A ., & Burger B . (2020). What comes first : combining motion capture and eye tracking data to study the order of articulators in constructed action in sign language narratives. LREC Proceedings. European Language Resources Association, 18, 583–594. https://www.aclweb.org/anthology/2020.lrec-1.735.pdf

[bibr44-20416695221116652] Jongerius C. Callemein T. Goedemé T. Beeck Van K ., Romijn J. A ., Smets E. M. A ., & Hillen M. A . (2021). *Eye-tracking glasses in face-to-face interactions : Manual versus automated assessment of areas-of-interest* . 10.3758/s13428-021-01544-2 PMC8516759 33742418

[bibr45-20416695221116652] Kamali K. Akbari A. A. Desrosiers C. Akbarzadeh A. Otis M. J. Ayena J. C . (2020). Low-Rank and sparse recovery of human gait data. *Sensors (Switzerland)*, *20*(16), 1–13. 10.3390/s20164525 PMC7472490 32823505

[bibr46-20416695221116652] Kiefer P. Giannopoulos I. Raubal M. Duchowski A. (2017). Eye tracking for spatial research: cognition, computation, challenges. In Spatial cognition and computation, *17*(1–2), 1–19. 10.1080/13875868.2016.1254634

[bibr47-20416695221116652] Knudson D . (2017). Confidence crisis of results in biomechanics research. Sports Biomechanics, 16(4), 425–433. 10.1080/14763141.2016.1246603 28632059

[bibr48-20416695221116652] Kothari R ., Yang Z ., Kanan C ., Bailey R ., Pelz J. B ., & Diaz G. J . (2020). Gaze-in-wild: A dataset for studying eye and head coordination in everyday activities. Scientific Reports, 10(1), 1–18. 10.1038/s41598-020-59251-5 32054884 PMC7018838

[bibr49-20416695221116652] Kurzhals K. Hlawatsch M. Seeger C. Weiskopf D . (2017). Visual analytics for mobile eye tracking. IEEE Transactions on Visualization and Computer Graphics, 23(1), 301–310. 10.1109/TVCG.2016.2598695 27875146

[bibr50-20416695221116652] Laidlaw K. E. W. Foulsham T. Kuhn G. & Kingstone A . (2011). Potential social interactions are important to social attention. Proceedings of the National Academy of Sciences of the United States of America, 108(14), 5548–5553. https://doi.org/10.1073/pnas.1017022108 21436052 10.1073/pnas.1017022108 PMC3078350

[bibr51-20416695221116652] Lappi O. (2015). Eye tracking in the wild : The good, the bad and the ugly. *Journal of Eye Movement Research*, 8(5), 1–21. 10.16910/jemr.8.5.1

[bibr52-20416695221116652] Lee D. N. Lishman J. R. Thomson J. A . (1982). Regulation of gait in long jumping. Journal of Experimental Psychology: Human Perception and Performance, 8(3), 448–459. 10.1037/0096-1523.8.3.448

[bibr53-20416695221116652] MacInnes J. Iqbal S. Pearson J. Johnson E. N . (2018). Wearable eye-tracking for research: automated dynamic gaze mapping and accuracy/precision comparisons across devices. *BioRxiv* .

[bibr54-20416695221116652] Mardanbegi D. Hansen D. W . (2012). Parallax error in the monocular head-mounted eye trackers. Proceedings of the 2012 ACM Conference on Ubiquitous Computing, 689–694.

[bibr1054-20416695221116652] Marigold, D. S., & Patla, A. E. (2008). Visual information from the lower visual field is important for walking across multi-surface terrain. *Experimental Brain Research, 188*(1), 23–31. 10.1007/s00221-008-1335-7 18322679

[bibr55-20416695221116652] MathWorks Inc. (2019). MATLAB (No. 2019b).

[bibr56-20416695221116652] Matthis J. Yates J. Hayhoe M . (2018). Gaze and the control of foot placement when walking in natural terrain. Current Biology, 28(8), 1224–1233.e5. 10.1016/j.cub.2018.03.008 29657116 PMC5937949

[bibr57-20416695221116652] Mele M. L. Federici S . (2012). Gaze and eye-tracking solutions for psychological research. Cognitive Processing, 13(1), 261–265. 10.1007/s10339-012-0499-z 22810423

[bibr58-20416695221116652] Montagne G. Cornus S. Glize D. Quaine F. Laurent M . (2000). A perception-action coupling type of control in long jumping. Journal of Motor Behavior, 32(1), 37–43. 10.1080/00222890009601358 11008270

[bibr59-20416695221116652] Musial J. P. Verstraete M. M. Gobron N . (2011). Technical note : comparing the effectiveness of recent algorithms to fill and smooth incomplete and noisy time series. Atmospheric Chemistry and Physics, *11*(15), 7905–7923. 10.5194/acp-11-7905-2011

[bibr60-20416695221116652] Navia J. A. Dicks M. van der Kamp J. Ruiz L. M . (2017). Gaze control during interceptive actions with different spatiotemporal demands. Journal of Experimental Psychology: Human Perception and Performance, 43(4), 783–793. 10.1037/xhp0000347 28345945

[bibr61-20416695221116652] Niehorster D. C. Hessels R. S. Benjamins J. S . (2020). Glassesviewer: open-source software for viewing and analyzing data from the Tobii Pro Glasses 2 eye tracker. Behavior Research Methods, 52(3), 1244–1253. 10.3758/s13428-019-01314-1 31898293 PMC7280338

[bibr62-20416695221116652] Niehorster D. C. Santini T. Hessels R. S. Hooge I. T. C. Kasneci E. Nyström M . (2020). The impact of slippage on the data quality of head-worn eye trackers. Behavior Research Methods, 52(3), 1140–1160. 10.3758/s13428-019-01307-0 31898290 PMC7280360

[bibr63-20416695221116652] Niehorster D. C. Zemblys R. Beelders T. Holmqvist K . (2020). Characterizing gaze position signals and synthesizing noise during fixations in eye-tracking data. Behavior Research Methods, 52(6), 2515–2534. 10.3758/s13428-020-01400-9 32472501 PMC7725698

[bibr64-20416695221116652] Nyström M. Holmqvist K . (2010). An adaptive algorithm for fixation, saccade, and glissade detection in eyetracking data. Behavior Research Methods, 42(1), 188–204. 10.3758/BRM.42.1.188 20160299

[bibr65-20416695221116652] Ooms K . (2015). Accuracy and precision of fixation locations recorded with the low-cost Eye Tribe tracker in different experimental set- ups. Journal of Eye Movement Research, 8(1), 1–24. 10.16910/jemr.8.1.5

[bibr66-20416695221116652] Parr J. V. V. Foster R. J. Wood G. Thomas N. M. Hollands M. A . (2020). Children with developmental coordination disorder show altered visuomotor control during stair negotiation associated with heightened state anxiety. Frontiers in Human Neuroscience, 14(November) 1–15. 10.3389/fnhum.2020.589502 PMC7731582 33328936

[bibr67-20416695221116652] Pastel S. Hsi C. Luca C. Mats M. Katharina N. Kerstin P . (2020). Comparison of gaze accuracy and precision in real - world and virtual reality. Virtual Reality, 25(1), 175–189. 10.1007/s10055-020-00449-3

[bibr68-20416695221116652] Pinheiro J. Bates D. DebRoy S. Sarkar D. Core T. R. (2020). Nlme: linear and nonlinear mixed effects models (R package version 3.1-151,). https://cran.r-project.org/package=nlme%3E

[bibr69-20416695221116652] RStudio Team. (2020). RStudio: Integrated Development for R.

[bibr70-20416695221116652] Spörri J. Schiefermüller C. Müller E . (2016). Collecting kinematic data on a ski track with optoelectronic stereophotogrammetry: A methodological study assessing the feasibility of bringing the biomechanics lab to the field. PLoS ONE, 11(8), 1–12. 10.1371/journal.pone.0161757 PMC4999283 27560498

[bibr71-20416695221116652] Summan R. Pierce S. G. Macleod C. N. Dobie G. Gears T. Lester W . (2015). Spatial calibration of large volume photogrammetry based metrology systems. Measurement, 68, 189–200. 10.1016/j.measurement.2015.02.054

[bibr72-20416695221116652] Tatler B. W. Hayhoe M. M. Land M. F. Ballard D. H . (2011). Eye guidance in natural vision: Reinterpreting salience. Journal of Vision, 11(5), 5. 10.1167/11.5.5 PMC3134223 21622729

[bibr73-20416695221116652] Tatler B. W. Tatler S. L . (2013). The influence of instructions on object memory in a realworld setting. Journal of Vision, 13(2), 1–13. 10.1167/13.2.5 23390319

[bibr74-20416695221116652] Thibeault M. Jesteen M. Beitman A . (2019). Improved accuracy test method for mobile eye tracking in usability scenarios. Proceedings of the Human Factors and Ergonomics Society Annual Meeting, 63(1), 2226–2230. 10.1177/1071181319631083

[bibr75-20416695221116652] Tobii. (2018). Tobii Pro Glasses 2 Product Description. www.tobiipro.com

[bibr76-20416695221116652] van Andel S. Cole M. H. Pepping G. J . (2018). Perceptual-motor regulation in locomotor pointing while approaching a curb. Gait and Posture, 60, 164–170. 10.1016/j.gaitpost.2017.12.006 29241099

[bibr77-20416695221116652] van der Kamp J. Dicks M . (2017). Looking further! The importance of embedding visual search in action. The Behavioral and Brain Sciences, 40(April), e158. 10.1017/S0140525X16000273 29342623

[bibr78-20416695221116652] van der Kruk E. Reijne M. M . (2018). Accuracy of human motion capture systems for sport applications; state-of-the-art review. European Journal of Sport Science, 18(6), 806–819. 10.1080/17461391.2018.1463397 29741985

[bibr79-20416695221116652] van Dijk L. Bongers R. M . (2014). The emergence of an action system: the organization of gaze in creating novel tools. Ecological Psychology, 26(3), 177–197. 10.1080/10407413.2014.929476

[bibr80-20416695221116652] Vansteenkiste P. Cardon G. Philippaerts R. Lenoir M. (2014). *Measuring dwell time percentage from head-mounted eye-tracking data – comparison of a frame-by-frame and a fixation-by-fixation analysis* . December. 10.1080/00140139.2014.990524

[bibr81-20416695221116652] Vickers J. N . (1996). Visual control when aiming at a far target. Journal of Experimental Psychology: Human Perception and Performance, 22(2), 342–354. 10.1037/0096-1523.22.2.342 8934848

[bibr82-20416695221116652] Wang D. Mulvey F. B. Pelz J. B. Holmqvist K . (2017). A study of artificial eyes for the measurement of precision in eye-trackers. Behavior Research Methods, *49*(3), 947–959. 10.3758/s13428-016-0755-8 27383751

[bibr83-20416695221116652] Willmott C. J. Matsuura K . (2005). Advantages of the mean absolute error (MAE) over the root mean square error (RMSE) in assessing average model performance. Climate Research, 30(1), 79–82. 10.3354/cr030079

[bibr84-20416695221116652] Winter D. A. (2009). Biomechanics and motor control of human movement: fourth edition. In Biomechanics and motor control of human movement: Fourth edition (Fourth), 1–370. Wiley. 10.1002/9780470549148

[bibr85-20416695221116652] Young W. R. Wing A. M. Hollands M. A . (2012). Influences of state anxiety on gaze behavior and stepping accuracy in older adults during adaptive locomotion. Journals of Gerontology - Series B Psychological Sciences and Social Sciences, *67*(1), 43–51. 10.1093/geronb/gbr074 21808071

